# Lifestyle and Sociodemographic Parameters Associated with Mental and Physical Health during COVID-19 Confinement in Three Ibero-American Countries. A Cross-Sectional Pilot Study

**DOI:** 10.3390/ijerph18105450

**Published:** 2021-05-19

**Authors:** Ilse Adriana Gutiérrez-Pérez, Pedro Delgado-Floody, Daniel Jerez-Mayorga, Diego Soto-García, Felipe Caamaño-Navarrete, Isela Parra-Rojas, Nacim Molina-Gutiérrez, Iris Paola Guzmán-Guzmán

**Affiliations:** 1 Faculty of Chemical-Biological Sciences, Universidad Autónoma de Guerrero, Chilpancingo 39087, Mexico; q.ilsegtz@gmail.com (I.A.G.-P.); iselaparra@uagro.mx (I.P.-R.); 2 Department of Physical Education, Sport, and Recreation, Universidad de La Frontera, Temuco 4780000, Chile; pedro.delgado@ufrontera.cl; 3 Faculty of Rehabilitation Sciences, Universidad Andres Bello, Santiago 7591538, Chile; daniel.jerez@unab.cl; 4 Faculty of Sciences and Physical Activity and Sports, University of León, 24007 León, Spain; dsotg@unileon.es; 5 Faculty of Education, Universidad Católica de Temuco, Temuco 4780000, Chile; marfel77@gmail.com; 6 Department of Physical Therapy, Faculty of Health Sciences, Universidad Católica de Maule, Talca 3460000, Chile; nacim.molina@gmail.com

**Keywords:** lifestyle, mental health, physical activity, comorbidities

## Abstract

Background: The aim of the present study was to determine the association between the health-related quality of life (HRQoL) with sociodemographic parameters and lifestyle during COVID-19 confinement in Mexico, Chile, and Spain. Methods: A cross-sectional pilot study, with 742 observations of online surveys in 422, 190, and 130 individuals from Mexico, Chile, and Spain, respectively. Sociodemographic data, presence of comorbidities, food habits, and physical activity (PA) patterns were evaluated. The HRQoL was evaluated according to the SF-36 Health Survey. The multilinear regression analysis was developed to determine the association of variables with HRQoL and its physical and mental health dimensions. Results: The female sex in the three countries reported negative association with HRQoL (Mexico: β −4.45, *p* = 0.004; Chile: β −8.48, *p* < 0.001; Spain: β −6.22, *p* = 0.009). Similarly, bad eating habits were associated negatively with HRQoL (Mexico: β −6.64, *p* < 0.001; Chile: β −6.66, *p* = 0.005; Spain: β −5.8, *p* = 0.032). In Mexico, PA limitations presented a negative association with HRQoL (β −4.71, *p* = 0.011). In Chile, a sedentary lifestyle (h/day) was linked negatively with HRQoL (β −0.64, *p* = 0.005). In Spain, the highest associations with HRQoL were the presence of comorbidity (β −11.03, *p* < 0.001) and smoking (β −6.72, *p* = 0.02). Moreover, the PA limitation in Mexico (β −5.67, *p* = 0.023) and Chile (β −9.26, *p* = 0.035) was linked negatively with mental health. Conclusions: The bad eating habits, PA limitations, female sex, comorbidity presence, and smoking were parameters linked negatively with HRQoL.

## 1. Introduction

The coronavirus disease 2019, called COVID-19, was declared a pandemic by the World Health Organization (WHO) on 11 March 2020. Its evolution up to the time of writing this article (11 March 2021) has led to 118,006,153 cases diagnosed and 2,619,676 deaths, globally. The epidemiological panorama in Spain is 3,164,982 diagnosed cases and 71,727 deaths, in Chile 864,064 diagnosed cases and 21,182 deaths; and in Mexico 2,137,884 diagnosed cases and 191,789 deaths [1]. COVID-19 has resulted in a significant number of psychological consequences [2] and has affected the populations of all countries in terms of labor, economy, productivity, health, and consequently, people’s lifestyles. It has led to strict decisions regarding control, with physical distancing and limiting mobility as the main prevention measures [3,4]. Unfortunately, these actions bring with them a low level of physical activity (PA) [5]. Failure to comply with PA recommendations for adults, even during the COVID-19 outbreak [6] (i.e., 150 min of moderate-intensity physical activity throughout the week, or at least 75 min of vigorous-intensity, or be active at home for least 30 min per day), can lead to the functional and structural deterioration of the organism [7], decreasing mental and physical health [8,9]. During the COVID-19 confinement, an increase in sedentary activities has been reported, such as watching TV, using electronics, and logging into social media [10]. These negative changes in lifestyle habits, including change in diet, as well as more time in mentally passive sitting at home, are associated with higher odds of mentally ill health [11], as well as with different cardiometabolic risks [12,13,14].

Privacy and loneliness, corroborated in studies with people under quarantine, are associated with fear, anguish, general psychological symptoms, irritability, insomnia, and increased emotional or mental and mood disorders, such as anxiety and depression [15]. The evidence has shown that the population has had less PA during the COVID-19 pandemic [16]. This is unfortunate because a fundamental benefit of PA is the improvement of mental health, due to its anxiolytic and antidepressant effects, and it also protects and increases resilience against the physical and mental consequences of psychosocial stress during confinement [5,17]. A recent study in a Latin-American population reported the importance of maintaining PA during confinement [13] due to the fact that regular exercise and/or PA have been proved to elicit a wide range of health-promoting effects, bearing preventive, therapeutic, and sometimes even reverting actions, in a multitude of diseases [2,18].

Ibero-American countries have imposed limitations on the free movement of people, whereby social distancing can decrease the health-related quality of life (HRQoL) of the general population and especially of at-risk groups. Therefore, anyone who experiences confinement may have their HRQoL adversely affected, even to a small degree [19]. This is reflected in the number of online posts talking about depression, anxiety, and increased indignation, while the expression of positive emotions has decreased significantly [2]. Moreover, the negative changes in lifestyle may affect the mental and physical health of the population. Consequently, an investigation into the effects of the pandemic restrictions on HRQoL (i.e., physical and mental health dimension) can be considered a global public health need. The objective of the present study was to examine the physical and mental health dimensions and their association with sociodemographic parameters and lifestyle during COVID-19 confinement in three Ibero-American countries.

## 2. Materials and Methods

A cross-sectional study was performed. The sample comprised a total of 742 participants that were from Mexico (*n* = 422), Chile (*n* = 190), and Spain (*n* = 130). The data of 16 surveys were excluded from analysis due to incomplete information. The study was done in accordance with the Basics of Good Clinical Practice and the Declaration of Helsinki (2013) and was approved by the local Ethics Committees (Project identification code: CB-007/2K20 and ACTA N°145/2020).

### 2.1. Self-Report Measures

The information was collected by an electronic survey designed by a multidisciplinary team uploaded to the Google Forms platform. Previous to data collection, a pilot sampling was made of 35 people to evaluate problems of design and response collection (i.e., in the first week of June 2020). Once the survey had been tested and validated, it was shared by e-mail, Facebook, Instagram, and WhatsApp, for six weeks (from 20 June to 31 July 2020), during the first wave by COVID-19. In this period, Mexico [20] and Chile [21] had a strict “stay at home” message and closed-down of non-essential activities, meanwhile, Spain experimented with the first official end of lockdown (21st June) and mobility recovered under the health safety guidelines of “new normality” after 100 days of confinement [22]. The survey was circulated in Mexico, Chile, and Spain. Adults (aged ≥ 18 years) of both genders were invited to participate in the study using a snowball sampling method. The questionnaire was distributed anonymously, the participants were informed about the objectives of the study and consent electronically prior to filling the questionaries. The survey was divided into four sections, including informed consent, sociodemographic data, SF-36 questionnaire, food habits, and PA patterns.

### 2.2. Study Presentation and Informed Consent

In this section, the details of the research were presented. On the first page of the online questionnaire, study participants were asked to give their consent to participate before being guided to the questionnaire. Only after participants gave informed consent were they able to continue to the next pages. The responses were anonymous and confidential. The participants were free to leave the survey at any stage before the submission process. Responses were only accepted and considered in data processing when ‘submit’ was selected.

### 2.3. Sociodemographic and Anthropometric Data

The second section was related to sociodemographic data; sex (female, male); age (years); education level (primary, secondary school, university degree, and postdoctoral degree); current occupation (classified as: Employment, Housework, Student, Own business, Non-employment, and Retired); confinement level (Strict confinement, partial confinement, Non-confinement due to work (persons that because of a specific job situation couldn’t comply with confinement measures); released of home confinement (with mobility recovered under the health safety guidelines of “new normality”)); marital status (single, married, common-law married, separated, widowed); body weight (kg); and height (m). Body mass index (kg/m^2^) and its classification category (normal weight, overweight, or obese) were calculated. In this section, the presence of comorbidities was evaluated in a free question; ‘comorbidity’ was defined by the presence of one or more chronic diseases, classified in cardiometabolic-based, autoimmune, musculoskeletal, respiratory, or gastrointestinal disease.

### 2.4. Evaluation of Health-Related Quality of Life (HRQoL)

HRQoL was measured according to the SF-36 questionnaire. The SF-36 Health Survey is a 36-item self-report survey that assesses eight domains related to physical and mental health. It has 36 questions and generates a health profile of eight domains (subscales): physical functioning (PF); role limitations due to physical problems (physical role (PR); bodily pain (BP); general health (GH) perceptions; vitality (VT); social functioning (SF); role limitations due to emotional problems (emotional role (ER) and emotional well-being (EWB). Four domains (PF, RP, BP, and GH) constitute physical health and the other four domains (VT, SF, RE, and EWB) constitute mental health (MH) [23]. In addition, the SF-36 can be used to detect the amount of change in participants’ general health over the past year. The scores on all the domains are transformed into a scale from 0 to 100, where the highest score indicates the optimal HRQoL and the lowest score indicates the poorest HRQoL.

### 2.5. Lifestyle: Food Habits and Physical Activity (PA) Patterns

In this section, questions about eating habits and physical parameters, and consumption of tobacco or vaping and of alcohol (times per week) were asked. In general, the participants were asked about changes in their lifestyle behavior due to confinement by COVID-19. The general perception of diet was consulted (good, regular, or bad); and frequency of water consumption (glasses per day (250 mL)), as well as soda (units (500 mL) per day). Regarding PA, we asked whether the participants exercised (yes or no); whether they perceived their physical activity practice as being limited during confinement (PA limitation); the grade of limitation on a scale of 0–100 (PA limitation (scale)); the frequency of PA; sessions per week, times per week, and the number of minutes or hours spent per session. Sedentary behavior was defined according to the number of hours (h) or minutes per day sitting or reclining.

### 2.6. Data Analyses

Descriptive data are presented as mean (SD), range, and percentage. A Chi-square test was used for gender differences between proportions, and an ANOVA test was used to identify significant differences of mean among groups, and the Bonferroni test to compare within groups. Within each group, a logistic regression analysis was made to determine the effect (β coefficient), as well as odds ratio (OR), to determine the association between HRQoL (i.e., physical and mental health dimension) and sociodemographic parameters and lifestyle (i.e., eating habits and physical activity and sedentary behaviors). Scores for each dimension of the SF-36 were used as dependent variables one at a time. The independent variables were sociodemographics, nutritional habits, and physical parameters in models adjusted by age. In this study, for the total evaluated sample, the median for physical health was 77.8 score, meanwhile, for mental health, it was 73.2 score. We considered using the high quartile on a scale from 0–100 as a reference category on the association models. Therefore, HRQoL scores were binned into “poor” (<75 score) and “normal” (≥75 score), *p*-values of < 0.05 were accepted as significant. STATA V.13.0 (StatCorp College Station, TX, USA) was used for the statistical analyses.

## 3. Results

### 3.1. Sociodemographic Data and Presence of Comorbidities

According to sociodemographic characteristics, the sample from Mexico had a higher prevalence of university education (57%, *p <* 0.001) than Chile (43.7%) and Spain (43.9%), while Spain reported the highest prevalence in employed participants (61.5%, *p =* 0.003). The confinement level was similar between Mexico and Chile but different for Spain, given that the regions and countries showed a heterogeneous evolution in the incidence of COVID-19. No differences were observed in age, sex, marital status, or comorbidities (Table 1). In general, the principal self-report diseases were cardiometabolic-based chronic disease 15% (15.6% for Mexico, 12.6% for Chile and 16.2% for Spain), followed by asthma 5.0% (3.3%, 7.4%, and 6.9%, respectively); thyroid disease 2.3% (1.4%, 4.2%, 2.3%, respectively); endogenous depression 2.4% (1.7%, 5.3%, and 0.8%, respectively); gastrointestinal disease 2.02% (1.7%, 3.2%, and 1.5%); and rheumatoid arthritis 1.9% (1.9%, 2.6%, and 0.8%, respectively).

### 3.2. Physical Activity, Anthropometric and Food Habits Parameters

According to the anthropometric parameters, the participants of Spain reported taller heights than those in Chile and Mexico (*p* < 0.001). In relation to a sedentary lifestyle, the Chilean population reported the highest sedentariness when compared to Mexico (Table 2).

The smoker prevalence and alcohol consumption were significantly different between countries. Particularly, Spain reported higher alcohol consumption than Mexico. Although the soda consumption was similar between groups, the Chilean population reported the lowest number of glasses of water drunk per day (Table 3).

### 3.3. Associations between HRQoL and Dimensions by Countries

**Figure 1:**
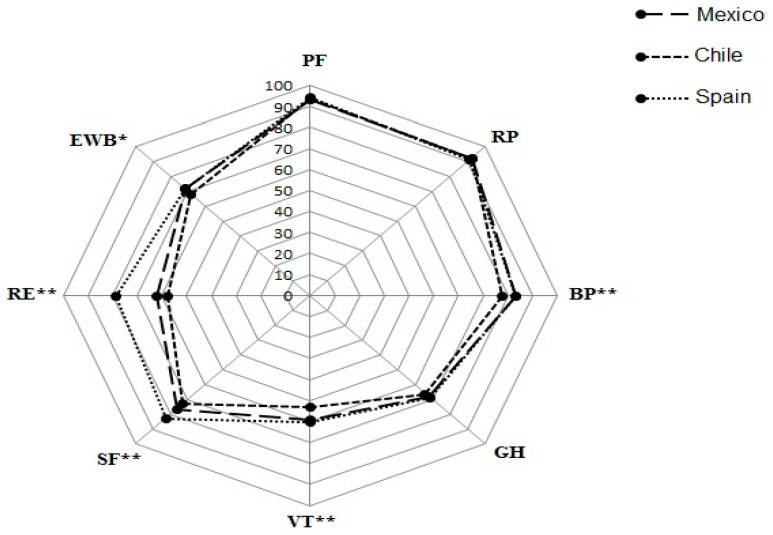
Health-related quality of life dimensions according to countries. PF, Physical functioning; RP, role limited by physical problems; BP, bodily pain; GH, general health; VT, vitality; SF, social functioning; RE, role limited by emotional problems; EWB, emotional well-being. * *p*-value < 0.05 and ** *p*-value ≤ 0.01.

The total score for HRQoL was different between countries (*p =* 0.002); Chile reported a lower HRQoL score compared with Spain. According to dimensions of health, the total score for mental health was higher in the Spanish population and lowest in the Chilean population. The emotional role dimension was decreased in Chile and Mexico, moreover, a diminished vitality was observed in the Chilean group. The social functioning was higher in Spain. Emotional well-being and bodily pain were lower in Chile in comparison to the Mexican population. The Spanish population showed a greater health change (Table 4 and visual data are show in Figure 1).

### 3.4. Associations between HRQoL and Mental and Physical Function with Sociodemographic Variables and Lifestyle

The multilinear regression analysis was developed to determine the association of variables with HRQoL and its physical and mental health dimensions. The female sex in the three countries reported negative association with HRQoL (Mexico; β −4.45, *p* = 0.004, Chile; β −8.48, *p* < 0.001, Spain; β −6.22, *p* = 0.009). Similarly, bad/poor eating habits were associated negatively with HRQoL (Mexico; β −6.64, *p* < 0.001, Chile; β −6.66, *p* = 0.005, Spain; β −5.8, *p* = 0.032). In Mexico (β −4.71, *p* = 0.011), PA limitations presented an inverse association with HRQoL. In Chile, a sedentary lifestyle (h/day) was linked negatively with HRQoL (β −0.64, *p* = 0.005). In Spain, the highest associations with HRQoL were the presence of comorbidities (β −11.03, *p* < 0.001) and smoking (β −6.72, *p* = 0.02) (Table 5).

Regarding the main variables related to mental health, we found that bad eating habits in Mexico (β −7.56, *p* < 0.001), Chile (β −9.64, *p* = 0.003), and Spain (β −8.43, *p* = 0.019) reported a negative association with mental health. Moreover, PA limitations present in Mexico (β −5.67, *p* = 0.023) and Chile (β −9.26, *p* = 0.035) were linked negatively to mental health. In Spain, comorbidities (β −11.03, *p* < 0.001) and being a smoker (β −6.72, *p* = 0.02) were the principal parameters related to poor scores in mental health (Table 5).

In general, in Chile and Mexico, the female sex, bad eating habits, and physical parameters were associated with “poorer” (<75 scores) physical and mental health than in the Spanish population. Meanwhile, in Spain, the presence of comorbidity was the principal variable associated (Table 6).

## 4. Discussion

The objective of the present study was to examine the HRQoL based on the evaluation of physical and mental health dimensions, and their association with socio-demographic parameters and lifestyles during COVID-19 confinement in three Ibero-American countries. The main results were: (i) the female sex in the three countries was linked in a negative way to HRQoL; (ii) lifestyle, such as PA limitations and bad/poor eating habits reported a negative effect on mental and/or physical health in the three countries; (iii) in Chile, a sedentary lifestyle presented a negative association with HRQoL; (iv) the presence of comorbidities was linked to physical and mental health in the Spanish population.

In the three countries (Mexico, Chile, and Spain), the female sex was linked negatively to HRQoL in mental and physical health dimensions. Similarly, in Austria, it has been found that symptoms of depression and anxiety during the pandemic made quarantine particularly stressful for adults under 35 years of age and specifically for the female sex [24]. In Cyprus, a negative impact on the quality of life is associated with the female sex (between 18 and 29 years), in addition to having a greater risk of suffering symptoms of depression and anxiety [25]. Likewise, in the Mexican population, greater psychological stress during confinement was positively correlated with being a young, employed woman and being single, among other variables [26]. Nevertheless, negative behavior such as PA limitations and sedentary lifestyles had a negative effect on HRQoL (i.e., physical or mental health). Long periods of confinement can generate fear, discouragement, and distress in both men and women. A recent review of mental health and COVID-19 reflects increased symptoms of anxiety and depression (16–28%), and self-reported stress (8%) during the pandemic [15]. Moreover, in Canada, the female sex performed fewer steps and moderate-to-vigorous PA than men [27], a situation that may influence mental and physical health. These results are in line with our results where the female sex in the three countries was linked negatively with HRQoL. Furthermore, other factors such as the workload increase related to their children’s schoolwork while maintaining a household and taking care of their family could be related.

Moreover, similar to our study, people who are physically inactive during confinement have lower overall scores in the psychological, social, and environmental domains of quality of life [28]. In this sense, feedback is sustained. The confinement and social distancing are associated with changes in lifestyle such as PA limitations and an increase in sedentary behavior [29]. Movement restrictions have made some forms of PA more difficult to achieve and have increased the likelihood of more sedentary behavior, moreover, sedentary behavior can have a negative impact on mental health [18]. In our study, PA limitations were associated with poor mental health, additionally, low vitality and role emotional scores predominated between Mexican and Chilean populations, countries under a greater confinement level at the time of the sampling. A recent study proved that home isolation during COVID-19 has had mixed influences on health and behaviors, where PA participation during confinement has been linked to better well-being and sleep quality in the Chinese population [29]. In a similar vein, another study reported that PA adherence to the World Health Organization (WHO) guidelines during COVID-19 confinement was associated with better levels of mental health in Spanish adults [30]. In addition, while isolation is a necessary measure to protect public health, a study concluded that this action could alter PA levels and eating behaviors in a health-compromising direction [31]. In this context, MC Dowell et al. [32] showed that higher levels of PA are associated with lower odds of anxiety in Irish adults. In addition, this study reported that moderate and high PA were associated with lower odds of anxiety compared with low PA. Similar to our results, a recent study indicated that a perceived decrease in PA was associated with poorer mental health during the COVID-19 confinement [33]. Likewise, the findings in another study reported that no longer meeting PA guidelines and increased screen time were associated with worse subjective well-being [34]. Maugeri et al. [35] suggested that a reduction of PA levels had a profound negative impact on psychological health and well-being in Italian subjects, therefore, it has been reported that maintaining regular PA in a safe home environment is an important strategy for healthy living during the COVID-19 crisis [5]. Unfortunately, there is strong evidence for a decrease in PA during the COVID-19 quarantine [36,37].

Bad/regular eating habits presented a negative association with HRQoL. Similarly, to our results, a study reported that subjects with healthy eating habits presented a higher HRQoL. This study also showed that eating home-cooked meals and eating breakfast is a protective factor for HRQoL in each domain [38]. In addition, it has been reported that healthy food habits are positively related to emotional well-being [39]. In a similar manner, a recent study indicated that healthy dietary habits were negatively associated with depression and anxiety symptoms in Spanish and Greek participants during the COVID-19 outbreak [40]. Ingram et al. [41] indicated that restrictions during quarantine have been associated with poorer psychological well-being. Furthermore, the results of this investigation reported that a poorer diet was linked with a more negative mood. Similarly, a recent study reported that when subjects ate at home and focused more on the quality of their food and eating, it had positive influences on the HRQoL during COVID-19 confinement [29]. Another study showed that healthy eating habits were associated with a better subjective well-being [42]. In the same way, a recent study conducted with Chinese subjects reported that physical inactivity and unhealthy eating habits, such as infrequent vegetable intake, infrequent fruit intake, and often skipping breakfast, were associated with lower subjective well-being [43].

In our study, we found that in the Spanish population the presence of comorbidity and being a smoker had an inverse association with HRQoL in terms of physical and mental health. During COVID-19 confinement, in the Spanish population, there was a significant decrease of moderate-intensity PA in males and females with chronic conditions [44], results that may affect the HRQoL in a population with comorbidity. Likewise, in an observational study conducted in Croatia, it was reported that women who smoked a greater number of cigarettes had a lower frequency and duration of PA than men and were more likely to gain weight, factors that influence a decrease in quality of life [45]. On the other hand, the presence of comorbidity is closely related to the severity and progression of the COVID-19 disease and higher mortality; there is a higher mortality rate in smoking subjects compared to healthy subjects; however, the presence of some comorbidities (e.g., diabetes) and diabetes + smoking increases the risk of mortality through COVID-19 [46]. Therefore, it is essential to maintain optimal levels of PA, because they may be protective elements for health [12,13,14].

The main limitation of this study is its cross-sectional design; which makes conclusive cause-effect relations difficult, therefore, these factors should also be measured in a longitudinal study in the future to clarify the direction of the associations. Another limitation was the different sample sizes between countries, which confines the generalizability of the results. Moreover, details of the PA intensity were not evaluated. Additionally, there was inherent misinformation in self-reported survey research and self-reporting of comorbidities. The results presented could also be found regardless of the COVID-19 confinement. The main strength was that three countries participated in this study and the study provides results on physical and mental health in a moment related to confinement by COVID-19.

## 5. Conclusions

A negative lifestyle (i.e., bad eating habits, PA limitations, and sedentariness), and female sex were linked with negative physical and mental health. In general, in Chile and Mexico, the female sex has an association with physical health and mental health, while in the Spanish population, poor physical and mental health were associated mainly with the presence of comorbidity and being a smoker. Therefore, a healthy lifestyle that includes good eating habits and PA are particularly important since they could be protective factors against poor health status. Finally, the behavior of the COVID-19 pandemic in the world suggests the maintenance of a pandemic wave that will rise or fall over time, so the levels of confinement can be maintained according to policies established by each country. Therefore, it is important to consider the design and application of strategies that mitigate the potential negative effects on physical and mental health associated with confinement.

## Figures and Tables

**Table 1:** Sociodemographic characteristics of the sample study according to country.

Variable	Mexico *n* = 422	Chile *n* = 190	Spain *n* = 130	*p*-Value
Age, years ^a^	34.33 ± 12.0	32.66 ± 11.42	35.93 ± 12.26	0.051
Sex *n* (%) ^b^				0.078
Female	247 (58.5)	111 (58.4)	62 (47.7)	
Male	175 (41.5)	79 (41.6)	68 (52.3)	
Level of education *n* (%) ^b^				<0.001
Basic	9 (2.2)	15 (7.9)	15 (11.5)	
Technical	63 (14.9)	43 (22.6)	33 (25.4)	
University	241 (57.1)	83 (43.7)	57 (43.9)	
Postgraduate	109 (25.8)	49 (25.8)	25 (19.2)	
Work status *n* (%) ^b^				0.003
Employment	198 (46.9)	105 (55.3)	80 (61.5)	
Housework	29 (6.9)	4 (2.1)	5 (3.8)	
Student	115 (27.25)	63 (33.2)	27 (20.8)	
Own business	45 (10.7)	8 (4.2)	13 (10.0)	
Non-employment	28 (6.6)	6 (3.2)	4 (3.1)	
Retired	7 (1.7)	4 (2.1)	1 (0.1)	
Confinement level *n*(%) ^b^				<0.001
Strict confinement	236 (55.9)	103 (54.2)	2 (1.5)	
Partial confinement	137 (32.5)	51 (26.8)	5 (3.9)	
Non-confinement due to work	36 (8.5)	25 (13.2)	15 (11.5)	
Released of home confinement	13 (3.1)	11 (5.8)	108 (83.1)	
Marital status *n* (%) ^b^				0.14
Single	222 (52.6)	120 (63.2)	82 (63.1)	
Married	142 (16.35)	51 (26.8)	33 (24.4)	
Common law married	35 (8.3)	14 (7.4)	10 (7.7)	
Separated	23 (5.45)	5 (2.6)	5 (3.9)	
Comorbidity presence *n* (%) ^b^				0.058
No	311 (73.7)	122 (64.2)	92 (70.8)	
Yes	111 (26.3)	68 (35.8)	38 (29.2)	

Data shows ^a^ represent mean and standard deviation and ^b^ proportions. *p*-value < 0.05 is statistically significant.

**Table 2:** Anthropometric and physical parameters in the population evaluated.

Variable	Mexico [A] *n* = 422	Chile [B] *n* = 190	Spain [C] *n* = 130	*p*-Value	Differences between Specific Groups
Anthropometric Parameters					
Height (m) ^a^	1.65 ± 0.10	1.66 ± 0.09	1.72 ± 0.09	<0.001	A = B; A < C *; B < C *
Weight (kg) ^a^	71.9 ± 18.1	71.15 ± 14.7	75.1 ± 15.4	0.09	A = B; A = C; B = C
BMI (kg/m^2^) ^a^	26.1 ± 5.29	25.56 ± 4.4	25.3 ± 4.1	0.17	A = B; A = C; B = C
BMI category *n* (%) ^b^				0.07	
Underweight (<18.5)	8 (1.9)	5 (2.6)	0 (0)		
Normal weight (18.5–24.9)	191 (45.3)	90 (47.4)	75 (57.7)		
Overweight (25–29.9)	152 (36.0)	74 (39.0)	40 (30.8)		
Obesity (≥30)	71 (16.8)	21 (11.0)	15 (11.5)		
PA Patterns					
PA limitation (Yes, %) ^b^	334 (79.2)	161 (84.7)	118 (90.8)	0.006	
PA limitation (scale 0–100) ^a^	49.9 ± 30.4	54.8 ± 29.0	56.5 ± 29.4	<0.001	A < B *; A < C *; B = C
PA (times per week) ^a^	4.2 ± 1.67	3.55 ± 1.5	4.5 ± 1.6	<0.001	A = C; A > B *; C > B *
PA (min/session) ^a^	57.4 ± 33.1	62.8 ± 31.9	57.2 ± 26.4	0.22	A = B; A = C; B = C
PA (min/week) ^a^	254.34 ± 197.41	231.6 ± 161.5	267 ± 177.7	0.28	A = B; A = C; B = C
Sedentary lifestyle (h/day) ^a^	6.7 ± 4.9	8.2 ± 5.0	7.7 ± 4.3	0.003	A < B *; A = C; B = C
Sedentary lifestyle (h/day) ^b^				<0.001	
<4	93 (31.0)	31 (16.5)	17 (13.3)		
4–8	127 (42.3)	90 (47.9)	71 (55.5)		
>8	80 (26.7)	67 (35.6)	40 (31.2)		

Data shows ^a^ represent mean and standard deviation and ^b^ proportions. BMI; body mass index, PA; physical activity. *p*-value < 0.05 is statistically significant. * *p*-value < 0.05.

**Table 3:** Lifestyle parameters in the sample study.

Variable	Mexico [A] *n* = 422	Chile [B] *n* = 190	Spain [C] *n* = 130	*p*-Value	Differences between Specific Groups
**Lifestyle parameters**					
Drink glasses of water per day ^a^	5.7 ± 2.6	4.1 ± 2.1	7.1 ± 4.1	<0.001	A > B *; A < C *; B < C *
Glasses of water per day ^b^				<0.001	
0–2	23 (5.5)	49 (25.8)	8 (6.2)		
3–5	184 (43.6)	104 (54.7)	41 (31.5)		
6–10	207 (49.1)	36 (19.0)	69 (53.5)		
>10	8 (1.9)	1 (0.5)	12 (9.2)		
Drink soda beverage (units per day) ^a^	2.5 ± 3.0	2.1 ± 3.0	2.0 ± 2.3	0.25	A = B; A = C; B = C
Alcohol consumption (times per week) ^a^	1.04 ± 1.54	1.35 ± 1.69	1.63 ± 2.17	0.004	A = B; A < C *; B = C
Nutrition during confinement, perception ^b^				0.19	
Very good	69 (16.4)	27 (14.2)	27 (20.7)		
Good	204 (48.3)	88 (46.3)	69 (53.1)		
Regular	126 (29.9)	59 (31.1)	30 (23.1)		
Bad	23 (5.4)	16 (8.4)	4 (3.1)		
Smoker (Yes, %) ^b^	69 (16.3)	48 (25.26)	28 (21.54)	0.03	

Data shows ^a^ represent mean and standard deviation and ^b^ proportions. *p*-value < 0.05 is statistically significant. * *p*-value < 0.05.

**Table 4:** Comparison of health related to quality of life and dimensions according to country.

Variable SF-36	Mexico (A)	Chile (B)	Spain (C)	*p*-Value	Difference between Specific Groups
Total score	75.82 ± 15.54	72.7 ± 15.94	78.65 ± 13.58	0.002	A = B; A = C; B < C *
Total score Physical	78.26 ± 12.94	76.41 ± 12.85	78.54 ± 12.53	0.20	A = B; A = C; B = C
Total score Mental health	73.37 ± 20.9	68.98 ± 21.85	78.76 ± 18.05	<0.001	A > B *; B < C *; A < C *
HRQoL dimensions SF-36					
Physical functioning	93.38 ± 11.72	93.9 ± 12.51	94.34 ± 11.31	0.69	A = B; A = C; B = C
Role physical	92.65 ± 21.98	92.5 ± 21.81	91.15 ± 23.88	0.79	A = B; A = C; B = C
Role emotional	62.32 ± 43.22	57.54 ± 44.95	78.71 ± 35.22	<0.001	A< C *; A = B; B < C *
Vitality	59.21 ± 18.79	53.26 ± 20.71	60.07 ± 16.34	<0.001	A = C; B < A *; B < C *
Emotional wellbeing	71.79 ± 17.87	68.2 ± 17.59	71.6 ± 16.33	0.05	A > B *; A = C; B = C
Social functioning	76.08 ± 23.69	72.43 ± 24.03	82.46 ± 21.56	<0.001	A = B; A < C *; B < C *
Bodily pain	83.3 ± 18.94	77.77 ± 21.13	82.92 ± 20.25	0.004	A = C; B < A *; B = C
General health	67.80 ± 18.73	65.97 ± 18.76	68.61 ± 18.80	0.40	A = B; A = C; B = C
Health change	59.41 ± 22.95	56.71 ± 23.66	52.5 ± 22.19	0.009	A = B; A > C *; B > C *

Data shows represent mean and standard deviation. *p* value < 0.05 is statistically significant. * *p*-value < 0.05.

**Table 5:** Effect of Sociodemographic variables and lifestyle on HRQoL and mental and physical health dimensions.

Variable SF-36	Mexico	Chile	Spain
Total Score	β Coefficient (IC95%), *p*-Value	β Coefficient (IC95%), *p*-Value	β Coefficient (IC95%), *p*-Value
Age	0.03 (−0.09, 0.15), 0.60	0.28 (0.08, 0.47), 0.005	0.21 (0.02, 0.40), 0.029
Female sex	−4.45 (−7.44, −1.46), 0.004	−8.48 (−12.96, −4.0), <0.001	−6.22 (−10.83, −1.61),0.009
Comorbidity	−2.39 (−5.77, 0.97), 0.16	−3.34 (−8.09, 1.4), 0.16	−11.03 (−15.86, −6.2), <0.001
Smoking	−1.84 (−5.87, 2.17), 0.36	−1.57 (−0.83, 3.6), 0.55	−6.72 (−12.35, −1.08), 0.02
BMI	−0.26 (−0.54, 0.02), 0.06	0.18 (−0.32, 0.70), 0.47	−0.17 (−0.74, 0.40), 0.56
Bad/poor eating habits	−6.64 (−9.69, −3.59), <0.001	−6.66 (−11.25, −2.08), 0.005	−5.8 (−11.08, −0.51), 0.032
Sodas comsumption	0.13 (−0.46, 0.73), 0.66	0.32 (−0.44, 1.09), 0.40	−1.84 (−2.81, −0.87), <0.001
Alcohol comsumption	1.24 (0.09, −2.4), 0.03	0.11 (−1.24, 1.34), 0.87	0.25 (−0.82, 1.34), 0.63
Water comsumption	0.83 (0.15, 1.51), 0.017	−0.45 (−1.53, 0.61), 0.40	0.06 (−0.51, 0.63), 0.83
No exercise	−1.56 (−5.6, 2.47), 0.44	−3.66 (−8.76, 1.43), 0.15	−1.07 (−7.89, 5.74), 0.75
PA limitation, scale	−0.05 (−0.10, −0.004), 0.031	−0.02 (−0.09, 0.05), 0.60	−0.06 (−0.14, 0.01), 0.11
PA limitation (yes)	−4.71 (−8.35, −1.07), 0.011	−6.3 (−12.6, −0.007), 0.05	−0.12 (−8.29, 8.05), 0.97
PA (times per week)	1.57 (0.41, 2.69), 0.006	−1.03 (−2.82, 0.75), 0.25	0.93 (−0.68, 2.55), 0.25
PA (min per session)	0.01 (−0.04, 0.07), 0.57	0.04 (−0.04, 0.12), 0.34	0.07 (−0.016, 0.17), 0.10
PA (min per week)	0.006 (−0.003, 0.01), 0.20	−0.0002 (−0.01, 0.01), 0.97	0.01 (−0.002, 0.02), 0.09
Sedentary lifestyle (h/day)	0.02 (−0.33, 0.39), 0.87	−0.64 (−1.08, −0.19), 0.005	−0.51 (−1.06, 0.02), 0.06
Total physical health score			
Age	0.02 (−0.07, 0.13), 0.59	0.12 (−0.03, 0.29), 0.11	0.15 (−0.01, 0.33), 0.08
Female sex	−3.09 (−5.59, −0.59), 0.015	−3.69 (−7.40, 0.005), 0.05	−3.23 (−7.57, 1.09), 0.14
Comorbidity	−2.12 (−4.93, 0.68), 0.13	−3.74 (−7.55, 0.06), 0.054	−10.08 (−14.54, −5.61), <0.001
Smoker	−0.52 (−3.87, 2.82), 0.75	−0.63 (−4.88, 3.60), 0.76	−4.57 (−9.83, 0.67), 0.08
BMI	−0.31 (−0.55, −0.08), 0.007	0.05 (−0.36, 0.47), 0.79	−0.43 (−0.96, 0.09), 0.10
Bad/poor eating habits	−5.72 (−8.25, −3.18), <0.001	−3.68 (−7.42, 0.05), 0.05	−3.16 (−8.1, 1.77), 0.20
Sodas comsumption	0.02 (−0.48, 0.53), 0.92	0.20 (−0.41, 0.82), 0.50	−1.56 (−2.48, −0.65), 0.001
Alcohol comsumption	1.02 (0.03, 2.0), 0.042	0.14 (−0.95, 1.23), 0.80	0.85 (−0.14, 1.85), 0.09
Water comsumption	0.67 (0.09, 1.25), 0.02	−0.018 (−0.88, 0.84), 0.96	−0.19 (−0.73, 0.33), 0.46
No exercise	−1.99 (−5.41, 1.43), 0.25	−3.64 (−7.74, 0.45), 0.08	−5.14 (−11.42, 1.13), 0.10
PA limitation (scale)	−0.04 (−0.08, −0.0002), 0.049	0.01 (−0.04, 0.08), 0.54	−0.05 (−0.13, 0.016), 0.13
PA limitation (yes)	−3.75 (−6.78, −0.72), 0.015	−3.31 (−8.42, 1.79), 0.20	1.61 (−5.92, 9.15), 0.67
PA (times per week)	−0.04 (−0.08, −0.0002), 0.049	0.01 (−0.04, 0.08), 0.54	−0.05 (−0.13, 0.016), 0.13
PA (min per session)	0.03 (−0.01, 0.08), 0.18	0.02 (−0.04, 0.09), 0.44	0.05 (−0.03, 0.13), 0.21
PA (min per week)	0.008 (0.0004, 0.016), 0.04	−0.001 (−0.014, 0.011), 0.81	0.005 (−0.006, 0.017), 0.39
Sedentary lifestyle (h/day)	−0.005 (−0.31, 0.30), 0.97	−0.31 (−0.68, 0.04), 0.08	−0.39 (−0.90, 0.11), 0.12
Total mental health score			
Age	0.03 (−0.12, 0.20), 0.65	0.43 (0.16, 0.70), 0.002	0.26 (0.01, 0.51), 0.04
Female sex	−5.81 (−9.83, −1.78), 0.005	−13.27 (−19.34, −7.20), <0.001	−9.21 (−15.3, −3.12), 0.003
Comorbidity	−2.66 (−7.20, 1.87), 0.24	−2.94 (−9.46, 3.58), 0.37	−11.99 (−18.58, −5.39), <0.001
Smoker	−3.17 (−8.57, 2.23), 0.24	−2.50 (−9.71, 4.7), 0.49	−8.86 (−16.36, −1.37), 0.021
BMI	−0.20 (−0.58, 0.17), 0.29	0.32 (−0.38, 1.02), 0.37	0.09 (−0.06, 0.86), 0.80
Bad/poor eating habits	−7.56 (−11.69, −3.44), <0.001	−9.64 (−15.91, −3.38), 0.003	−8.43 (−15.44, −1.43), 0.019
Sodas comsumption	0.24 (−0.56, 1.04), 0.55	0.44 (−0.60, 1.50), 0.40	−2.12 (−3.42, −0.82), 0.002
Alcohol comsumption	1.47 (−0.07, 3.02), 0.06	0.08 (−1.78, 1.94), 0.93	−0.33 (−1.77, 1.1), 0.64
Water comsumption	0.98 (0.07, 1.89), 0.03	−0.89 (−2.37, 0.57), 0.23	0.32 (−0.44, 1.08), 0.40
No exercise	−1.13 (−6.53, 4.26), 0.67	−3.68 (−10.7, 3.34), 0.30	2.99 (−6.01, 12.0), 0.51
PA limitation (scale)	−0.06 (0.13, −0.0007), 0.047	−0.06 (−0.16, 0.04), 0.26	−0.073 (−0.17, 0.03), 0.17
PA limitation (yes)	−5.67 (−10.57, −0.77), 0.023	−9.26 (−17.91, −0.67), 0.035	−1.85 (−12.72, 9.0), 0.73
PA (times per week)	1.67 (0.12, 3.21), 0.03	−1.21 (−3.64, 1.21), 0.32	1.72 (−0.50, 3.96), 0.12
PA (min per session)	−0.00001 (−0.07, 0.07), 0.99	0.05 (−0.05, 0.16), 0.34	0.10 (−0.02, 0.23), 0.11
PA (min per week)	0.004 (−0.009, 0.017), 0.54	0.001 (−0.02, 0.023), 0.92	0.019 (−0.0005, 0.038), 0.056
Sedentary lifestyle (h/day)	0.06 (−0.42, 0.55), 0.79	−0.96 (−1.57, −0.35), 0.002	−0.63 (−1.35, 0.08), 0.08

Data shown represent β coefficient regression (confidential interval 95%). BMI; body mass index, PA; physical activity. *p*-value < 0.05 is statistically significant.

**Table 6:** Association between “poor” physical a mental health with sociodemographic parameters and lifestyle.

Variable SF-36	Mexico	Chile	Spain
Total Physical Health (<75 Score)	OR (CI 95%), *p*-Value	OR (CI 95%), *p*-Value	OR (CI 95%), *p*-Value
Female sex	1.81 (1.16–2.81), 0.008	1.77 (0.93–3.37), 0.07	1.99 (0.86–4.62), 0.10
Comorbidity presence	1.31 (0.77–2.24), 0.30	2.70 (1.39–5.26), 0.003	7.75 (3.08–19.47), <0.001
Smoker	1.07 (0.58–1.78), 0.94	0.86 (0.42–1.76), 0.69	2.07 (0.83–5.1), 0.11
Excess weight (OW and OB)	0.76 (0.51–1.13), 0.18	0.99 (0.55–1.78), 0.98	1.54 (0.67–3.51), 0.29
Eating Regular-Bad	1.87 (1.19–2.93), 0.006	1.77 (0.94–3.35), 0.075	1.31 (0.53–3.21), 0.55
Drunk ≤ 5 glasses of water per day	1.25 (0.83–1.84), 0.29	0.73 (0.34–1.51), 0.41	1.29 (0.57–2.92), 0.52
No exercise	1.36 (0.79–2.34), 0.26	2.16 (1.06–4.36), 0.032	1.28 (0.40–4.01), 0.67
PA limitation (Yes)	1.47 (0.85–2.54), 0.15	1.68 (0.67–4.22), 0.26	0.70 (0.19–2.53), 0.59
Sedentary lifestyle (<8 h/day)	0.82 (0.43–1.57), 0.56	1.73 (0.64–4.7), 0.27	6.57 (0.75–57.0), 0.08
**Total mental health (<75 score)**			
Female sex	1.72 (1.16–2.56), 0.007	2.48 (1.35–4.56), 0.003	1.87 (0.85–4.13), 0.11
Comorbidity presence	1.17 (0.71–1.92), 0.51	1.40 (0.74–2.66), 0.29	4.0 (1.71–9.47), 0.001
Smoker	1.15 (0.69–1.94), 0.57	0.99 (0.50–1.94), 0.98	2.07 (0.86–4.98), 0.10
Excess weight (OW and OB)	1.20 (0.78–1.85), 0.39	0.82 (0.44–1.52), 0.54	1.68 (0.71–3.97), 0.23
Eating Regular-Bad	2.13 (1.39–3.26), <0.001	1.44 (0.78–2.65), 0.24	1.03 (0.44–2.43), 0.93
Drunk ≤ 5 glasses of water per day	1.09 (0.74–1.61), 0.63	0.88 (0.41–1.86), 0.74	2.22 (1.01–4.87), 0.045
No exercise	0.96 (0.57–1.61), 0.88	1.95 (0.95–3.98), 0.067	0.71 (0.21–2.4), 0.59
PA limitation	1.66 (1.02–2.7), 0.039	3.49 (1.41–8.35), 0.006	0.96 (0.26–3.45), 0.95
Sedentary lifestyle (<8 h/day)	1.08 (0.58–1.95), 0.82	6.0 (2.20–16.39), <0.001	1.79 (0.41–7.75), 0.43

Data shown represent Odd ratio (confidential interval 95%). Model adjusted by age. *p*-value < 0.05 is statistically significant. OW; Overweight, OB; Obesity, PA; Physical activity.

## Data Availability

The data presented in this study are available on request from the corresponding author. The data are not publicly available due to ethical restrictions.
